# Platelet-Derived Growth Factor Receptor-β Antagonism Restores Morphine Analgesic Potency against Neuropathic Pain

**DOI:** 10.1371/journal.pone.0097105

**Published:** 2014-05-12

**Authors:** Courtney L. Donica, Yan Cui, Shanping Shi, Howard B. Gutstein

**Affiliations:** 1 Department of Anesthesiology, The University of Texas – MD Anderson Cancer Center, Houston, Texas, United States of America; 2 Department of Biochemistry and Molecular Biology, Genes and Development Graduate Program, The University of Texas – MD Anderson Cancer Center, Houston, Texas, United States of America; University of Nebraska Medical Center, United States of America

## Abstract

**Background:**

Chronic, intractable pain is a problem of pandemic proportions. Pain caused by nerve injuries (neuropathic pain) is extremely difficult to treat. For centuries, opiates such as morphine have been the first-line treatment for severe chronic pain. However, opiates are often ineffective against neuropathic pain, leaving few options for suffering patients. We previously demonstrated that platelet-derived growth factor- β (PDGFR-β) inhibition completely eliminated morphine tolerance. In these studies, we determined whether PDGFR-β inhibition could improve the effectiveness of morphine for neuropathic pain treatment.

**Results and Findings:**

Spinal nerve ligation was performed in male Sprague-Dawley rats. The clinically used PDGFR antagonist imatinib did not relieve mechanical pain in a nerve injury model as determined by Von Frey assay. Surprisingly, combining imatinib with a previously ineffective dose of morphine led to complete pain relief. Scavenging released PDGF-B also markedly augmented the analgesic effect of morphine.

**Conclusions:**

These findings suggest the novel hypothesis that PDGF-B released by injured nerves renders animals resistant to morphine, implying that PDGFR-β inhibition could potentially eliminate the tremendous suffering caused by neuropathic pain.

## Introduction

In the United States alone, chronic pain afflicts over 100 million people at an estimated yearly cost to society of over 500 billion dollars [1]. Throughout recorded history, opioid drugs such as morphine have been a mainstay of treatment for severe, chronic pain [2]. However, over time tolerance to opioid analgesia develops. Because there are few alternatives to opioids for the treatment of intractable pain, marked increases in opioid dose may be required to compensate for inadequate analgesia as tolerance develops. However, tolerance to the unpleasant or potentially life-threatening side effects of opioids such as respiratory depression, constipation, urinary retention and delirium, does not occur as rapidly as analgesic tolerance [2], [3]. Therefore, patients face increased risk as well as suffering when opioids lose effectiveness.

It has been proposed that pain and tolerance utilize common signaling mechanisms [4]. We recently discovered that the platelet derived growth factor beta (PDGFR-β) is a highly selective and specific mediator of morphine tolerance [5]. We established that PDGFR-β signaling is both necessary and sufficient to cause morphine tolerance, and that morphine induced the release of PDGF-B, which caused tolerance to occur. Pain due to nerve injury (neuropathic pain) is particularly resistant to opioids, although high doses of morphine can temporarily relieve neuropathic pain in rodents [6]–[8]. It is estimated that 40–60% of people suffering from neuropathic pain have inadequate pain relief [9]. It is thought that one of the most difficult features of neuropathic pain to treat is mechanical hypersensitivity caused by nerve injury [10]. We hypothesized that mechanical hypersensitivity could be resistant to the analgesic effects of morphine because the nerve injury itself induced analgesic tolerance. In an animal model of neuropathic pain [11], we found that not only did PDGFR-β inhibition block analgesic tolerance, but also markedly improved the analgesic effectiveness of morphine against mechanical hypersensitivity. Additional experiments suggested the hypothesis that PDGF-B release by injured nerves could render neuropathic pain resistant to the analgesic effects of morphine.

## Methods

### Animals

Male Sprague Dawley rats (175–200 g, Harlan) were housed in groups of three and were maintained on a 12 hr light/dark cycle with *ad libitum* access to food and water. Rats habituated to the colony room for one week prior to experimental manipulations. Left L5 spinal nerve ligations were performed as described [11]. All protocols were approved by the MD Anderson Cancer Center Institutional Animal Care and Use Committee.

### Drug Administration

Drugs were dissolved in a solution of 10% β-cyclodextrin sulfobutyl ether (Captisol®, CyDex, Lenexa, KS) solution and 0.45% saline. Morphine sulfate was obtained from the MD Anderson Cancer Center Pharmacy, imatinib from LC Laboratories (Woburn, MA) and recombinant human PDGFR-β-Fc from R&D Systems (Minneapolis, MN). PDGFR-β-Fc was re-constituted in phosphate buffered saline (PBS) with 0.1% bovine serum albumin (BSA) at 100 µg/mL and stored at −80°C until use. Drugs were administered daily via subcutaneous injection or lumbar puncture as previously described [12].

### Nociceptive Testing

Animals were placed in Plexiglas cages on a mesh surface and habituated for 30 min per day for 3 days prior to testing. Mechanical sensitivity was assessed by Von Frey filaments using the up-down method of Dixon and median 50% threshold determined as described [13], [14].

### Statistical Analyses

Data were analyzed using GraphPad v 5.0 and was considered statistically significant if P<0.05 by two-way analysis of variance (ANOVA).

## Results

**Figure 1:**
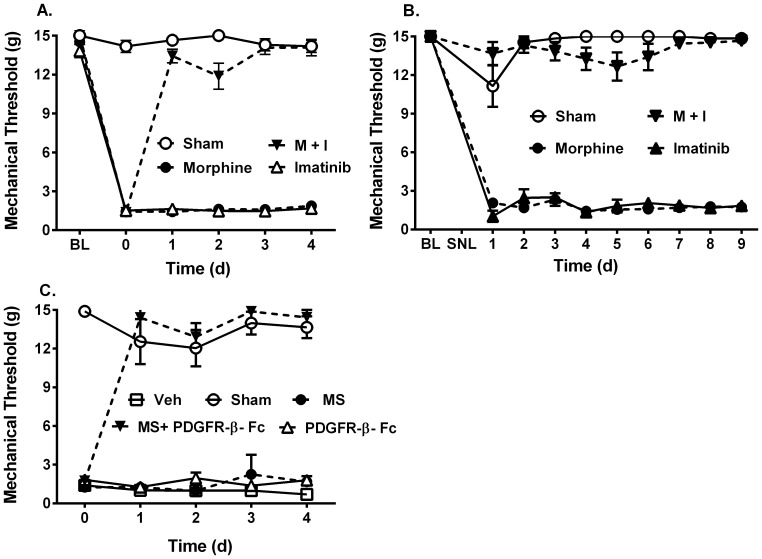
PDGFR inhibition restores morphine efficacy against neuropathic pain. Animals underwent left L5 SNL as described in [11]. Mechanical sensitivity was tested using von Frey filaments and 50% median response threshold determined [13], [14]. Sham operated animals underwent the same surgical procedure except a suture was not tied around the L5 nerve root. (**A**) Baseline mechanical sensitivity was determined before surgery (BL). Animals then underwent SNL and were allowed to recover for two weeks. Mechanical sensitivity was tested to confirm that SNL induced mechanical allodynia (day 0). SNL animals received daily intrathecal (i.t.) injections of morphine (2 nmol), imatinib (10 µg), or the combination and mechanical sensitivity was determined. Sham operated animals received injections of vehicle alone. Neither morphine nor imatinib alone were analgesic. Co-administration of morphine and imatinib completely reversed SNL-induced mechanical allodynia. n = 8–9 per group. (**B**) Beginning the day after SNL, animals received daily subcutaneous (s.c.) injections of morphine (2.5 mg/kg), imatinib (5 mg/kg), morphine + imatinib or vehicle and mechanical sensitivity determined. Systemic co-administration of morphine and imatinib completely eliminated allodynia after nerve injury. n = 5–9 per group. (**C**) Following a two week recovery after SNL, animals received daily i.t. injections of morphine (2 nmol), PDGFR-β-Fc scavenger (500 ng), the combination or vehicle. While PDGFR-β-Fc had no effect on mechanical allodynia, co-administration of morphine and PDGFR-β-Fc completely restored the effectiveness of morphine. n = 7–10 per group. All data presented as grams +/− s.e.m; all p<0.0001 (2-way ANOVA).

We initially administered morphine in the presence or absence of the PDGFR inhibitor imatinib [15] daily for 4 days in rats that underwent spinal nerve ligation (SNL). A morphine dose that is analgesic in thermal assays of nociception in non-ligated animals [5] (2 nmol, injected intrathecally (i.t.)) had no effect on mechanical hypersensitivity (Figure 1a). Imatinib alone also had no effect. Surprisingly, administering morphine and imatinib together completely eliminated the mechanical allodynia induced by SNL (Figure 1a; Treatment F_(3,31)_ = 1009, Day F_(5,155)_ = 339.2, Interaction F_(15,155)_ = 92.55; all p<0.0001 by two-way ANOVA).

We then determined whether imatinib would block mechanical allodynia from the initiation of nerve injury when given systemically. Animals were treated with a morphine dose that is analgesic in tests of thermal nociception (2.5 mg/kg, injected subcutaneously (s.c.)), imatinib (5 mg/kg, s.c.), the combination, or vehicle starting the day after SNL. Neither morphine nor imatinib alone altered mechanical hypersensitivity. Similar to the results obtained above after a two week surgical recovery (Figure 1a), combining morphine and imatinib produced immediate and complete reversal of mechanical allodynia from the day after nerve injury until the study was concluded 9 days later (Figure 1b; Drug F_(4,190)_ = 443.9, Day F_(4,190)_ = 11.13, Interaction F_(16,190)_ = 16.61; all p<0.0001 by two-way ANOVA).

These results suggested the hypothesis that release of PDGF by ligated nerves could render the resulting mechanical hypersensitivity resistant to the analgesic effects of morphine. To test this hypothesis, animals that had undergone SNL were treated with either 2 nmol morphine i.t., 500 ng of a fusion protein constructed of the extracellular domains of the PDGFR-β fused to antibody Fc fragments (PDGFR-β-Fc), which scavenges released PDGF-B (5), the combination, or vehicle. Administering vehicle, morphine, or PDGFR-β-Fc alone had no effect on mechanical hypersensitivity. Remarkably, combining morphine with PDGFR-β-Fc completely eliminated mechanical allodynia (Figure 1c; Treatment F_(4,29)_ = 186.9, Day F_(9,261)_ = 43.29, Interaction F_(36, 261)_ = 12.04; all p<0.0001 by two-way ANOVA). Similar to the combination of morphine and imatinib, repeated doses of PDGFR-β-Fc and morphine continued to block mechanical allodynia. These results support the hypotheses that 1) PDGF-B released by injured nerves reduces the effectiveness of morphine against mechanical allodynia and 2) that imatinib augments the effectiveness of morphine against mechanical allodynia by blocking PDGFR-β-mediated signaling.

## Discussion

Our findings demonstrate that PDGFR-β inhibition, either spinally or systemically, enhances morphine effectiveness against neuropathic pain. Combining a previously ineffective morphine dose with PDGFR-β antagonists can reverse established allodynia as well as prevent allodynia from developing after nerve injury. In addition, similar to our previously reported results [5], PDGFR-β inhibition prevents tolerance to this effect from developing. Interestingly, imatinib alone does not affect morphine analgesia [5]. Further, repeated administration of morphine with imatinib remains effective through at least 9 days (figure 1B). This suggests that there is no “tolerance” to the PDGFR-β antagonist rescue of morphine effectiveness. These results suggest that lower doses of opioids could be used to treat neuropathic pain if PDGFR-β inhibitors were given concurrently. In addition, the dose escalation that commonly occurs [9] could be avoided. Since tolerance to unpleasant or potentially life-threatening side effects of opioids such as respiratory depression, constipation, urinary retention and delirium, does not occur as rapidly as analgesic tolerance [3], PDGFR-β antagonism could potentially reduce the risks associated with chronic opioid treatment [16] and the suffering that results when opioids lose effectiveness.

We previously demonstrated that administration of PDGF-B to naïve animals rendered them tolerant to subsequent morphine doses [5]. PDGF-B is localized in the dorsal root ganglion and dorsal horn of the spinal cord and PDGFR-β is located in myelinated and unmyelinated nerves, dorsal root ganglion neurons and the spinal dorsal horn [17]–[20]. Given the distribution of PDGF-B and PDGFR-β, it is possible that PDGFR-β inhibition blocks opioid tolerance by modulating opioid effects on peptidergic primary afferent fibers. It has been suggested that pain and opioid tolerance utilize common signaling pathways [4], [21]. Our current observation that PDGF-B scavenging augmented the analgesic effect of morphine against neuropathic pain indicates that nerve ligation also induces PDGF-B release. PDGF-B alone does not appear to induce pain: PDGF-BB administration does not induce thermal sensitivity [5] and scavenging released PDGF-B alone does not alleviate neuropathic pain (Fig 1C). Rather, our data suggests that released PDGF-B affects the ability of morphine to induce analgesia. Taken together, these findings suggest that PDGF-B released by injured nerves causes resistance to the analgesic effect of morphine, possibly through similar translational and post-translational modifications of analgesic effectors induced by prior opioid exposure. In effect, PDGF-B renders animals “pre-tolerant” to morphine analgesia. Previous work has shown that neuropathic pain reduces the efficacy of morphine, supporting this hypothesis [6]–[8]. Our results also suggest that blockade of PDGFR-β signaling could become an important therapeutic approach for relieving the suffering of untold numbers of patients living with intractable neuropathic pain.
